# Immunological Heterogeneity

**DOI:** 10.1093/geroni/igaa057.3146

**Published:** 2020-12-16

**Authors:** Laura Haynes

**Affiliations:** UConn Health, Farmington, Connecticut, United States

## Abstract

Ease of access to circulating peripheral blood cells (PBMCs) can offer unique insights into human immune function, as well as responses to vaccination and infection. Nevertheless, PBMC heterogeneity has been under-appreciated since results obtained from mixed populations may reflect changes in subset abundance as opposed to true age-related changes involving a specific subset. Technological advances have allowed for the examination of age-related heterogeneity with regards to systemic cytokine levels, immune cell frequencies and chemokine receptor expression by peripheral lymphocytes. Furthermore, introduction of sex as a variable in the examination of human PBMCs adds additional dimorphism to the study of aging and immunity including differences in epigenetic modifications, levels of pro-inflammatory activity and adaptive immunity.

